# Stromal Vascular Cell-Mediated Inflammatory and Remodeling Pathways in Alcohol-Induced Visceral Adipose Tissue Dysfunction: A Pilot Study

**DOI:** 10.3390/biom16101440

**Published:** 2026-10-03

**Authors:** Weilun Ai, Arul Daniel John Kathiravan, Carol A. Casey, Sundararajan Mahalingam, Karuna Rasineni, Viswanathan Saraswathi

**Affiliations:** 1 Division of Diabetes, Endocrinology, and Metabolism, Department of Internal Medicine, University of Nebraska Medical Center, Omaha, NE 68198, USA; 2 VA Nebraska-Western Iowa Health Care System, Omaha, NE 68105, USA; 3 Division of Gastroenterology and Hepatology, Department of Internal Medicine, University of Nebraska Medical Center, Omaha, NE 68198, USA; 4 Department of Biochemistry and Molecular Biology, University of Nebraska Medical Center, Omaha, NE 68198, USA

**Keywords:** alcohol, visceral adipose tissue, stromal vascular cells, purinergic receptor, transforming growth factor

## Abstract

Chronic alcohol use induces adipocyte lipolysis and production of inflammatory mediators from adipose tissue (AT). Stromal vascular cells (SVCs) are a heterogeneous group of AT cells and contribute to AT inflammation and remodeling in metabolic syndrome. However, the role of SVCs in promoting AT dysfunction upon alcohol exposure remains unclear. Male rats were fed a Lieber–DeCarli diet containing 6.7% (*v*/*v*) ethanol (ET) or an isocaloric control diet (CON) for six weeks. Primary rat SVCs were isolated from visceral AT (VAT) of CON and ET rats. RNA sequencing analysis was performed to determine the overall changes in the gene-expression profile. Real-time PCR, Western blotting, and histological analyses were performed to validate the transcriptomics results. Chronic alcohol feeding decreased the adipocyte size and increased the formation of crown-like structures in the rat VAT. RNA-seq analysis revealed that signaling pathways related to cellular response to interleukin 1 (IL1) and collagen-containing extracellular matrix were significantly altered upon ethanol exposure in SVCs. Along these lines, ethanol exposure increased the protein levels of purinergic receptor P2Y2 (P2RY2) and IL1β in the SVCs of rat VAT. Furthermore, ethanol increased collagen deposition in both interstitial and perivascular regions, which was associated with an increase in the level of phosphorylated SMAD2/3 and collagen type VIII alpha 1 chain expression. These findings suggest that chronic ethanol exposure is associated with inflammatory and remodeling-related alterations in VAT and the total VAT-derived SVC fraction. This exploratory pilot study identifies P2RY2-associated inflammatory markers and TGFβ-related remodeling markers as candidates for future mechanistic investigation.

## 1. Introduction

Alcohol-associated liver disease (ALD) is one of the leading causes of chronic liver diseases and accounts for nearly 4% of all-cause mortality [1,2]. Chronic alcohol exposure exerts detrimental effects on adipose tissue (AT) by inducing oxidative stress, adipocyte lipolysis, and production of pro-inflammatory mediators, including interleukin 1β (IL1β), tumor necrosis factor α (TNFα), and monocyte chemoattractant protein-1 [3,4]. There is a tight link between visceral AT (VAT) inflammation and liver diseases. Pro-inflammatory cytokines are released from AT into circulation, which aggravate ethanol-induced liver lesions via the crosstalk between the AT and liver [4]. A clinical study of ALD individuals has reported an increase in the levels of interleukins and TNFα in AT, which is associated with elevated inflammatory markers in the liver [5].

Stromal vascular cells (SVCs) are a heterogeneous group of AT cells, including endothelial cells, fibroblasts, immune cells, and adipose stem cells, and contribute to AT inflammation and remodeling in diverse diseases [6]. For example, SVCs contribute to the production of pro-inflammatory mediators from AT in metabolic syndrome, promoting the development of insulin resistance [7]. The presence of inflammatory cytokines derived from SVCs stimulates excessive production of extracellular matrix (ECM) protein, which, in turn, leads to omental AT fibrosis [8]. In terms of ALD, chronic, multiple-binge alcohol exposure gives rise to adipose inflammation by promoting pro-inflammatory polarization of macrophages and increasing the generation of pro-inflammatory cytokines [9]. Moreover, chronic alcohol exposure exerts pro-inflammatory effects in AT by increasing macrophage infiltration into AT, which is associated with impairment of insulin-mediated glucose uptake in AT [10].

While chronic alcohol exposure is known to induce adipose tissue dysfunction, the contribution of SVCs to alcohol-induced AT inflammation and remodeling remains poorly defined. Here, the present pilot study examined pathological changes in VAT and VAT-derived SVCs to identify alcohol-associated transcriptional pathways that may contribute to adipose tissue inflammation and remodeling. We used primary SVCs collected from VAT of rats fed a chronic ethanol (6.7% *v*/*v*) diet for 6 wk.

## 2. Materials and Methods

### 2.1. Reagents

The reagents, chemicals, and supplies used in the current study include: collagenase II (C6885, Sigma-Aldrich, St. Louis, MO, USA), Fetal Bovine Serum (FBS, A5670801, Fisher Scientific, Waltham, MA, USA), Picro Sirius Red Stain Kit (Ab150681, Abcam, Cambridge, UK), mounting medium (SP15-100, Fisher Scientific), Tris-EDTA buffer for antigen retrieval (pH 9.0, ab93684, Abcam), Tris-buffered saline with Tween-20 (TBST, sc-362311, Santa Cruz Biotechnology, Dallas, TX, USA), rabbit-specific horseradish peroxidase/diaminobenzidine immunohistochemistry detection kit (ab64261, Abcam), TRIzol reagent (Ambion Life Technologies, MA, USA), NuGEN Universal Plus mRNA-Seq library kit (TECAN Genomics, Redwood City, CA, USA), 5× iScript reverse transcription supermix (1708841, Bio-Rad, Hercules, CA, USA), iQ supermix (1708862, Bio-Rad), Taqman^TM^ real-time PCR primers (Applied Biosystems, Waltham, MA, USA), including *P2ry2* (Rn02070661_s1), D-box binding PAR BZIP transcription factor (*Dbp*, Rn01498425_m1), collagen type VIII alpha 1 chain (*Col8a1*, Rn07363068_m1), ELOVL fatty acid elongase 6 (*Elovl6*, Rn00592815_m1), glyceraldehyde-3-phosphate dehydrogenase (*Gapdh*, Rn01775763_g1), acyl-CoA synthetase long chain family member 3 (*Acsl3*, Rn00589037_m1), sterol regulatory element binding transcription factor 2 (*Srebf2*, Rn01502638_m1), RT1 class II, locus DOa (*Rt1-DOa*, Rn00788273_m1), RT1 class II, locus Bb (*Rt1-Bb*, Rn01429090_g1), neuronal PAS domain protein 2 (*Npas2*, Rn01438223_m1), farnesyl-diphosphate farnesyltransferase 1 (*Fdft1*, Rn00570323_m1), antibodies against IL1β (26048-1-AP, Proteintech, Rosemont, IL, USA), P2RY2 (NBP3-12229, Novus Biologicals, Centennial, CO, USA), leukocyte common antigen (CD45, ab10558, Abcam), tumor necrosis factor α (TNFα, sc-52746, Santa Cruz Biotechnology), COL8A1 (17251-1-AP, Proteintech), SMAD family member 2 and 3 (SMAD2/3, 8685, Cell Signaling Technology, Danvers, MA, USA), phosphorylated SMAD2/3 (8828, Cell Signaling Technology), transforming growth factor β (TGFβ, sc-52893, Santa Cruz Biotechnology), anti-rabbit secondary antibody conjugated to horseradish peroxidase (7074, Cell Signaling Technology), anti-mouse StarBright^TM^ Blue 520 fluorescence secondary antibody (12005867, Bio-Rad, Hercules, CA, USA), and enhanced chemiluminescence reagent (1705062, Bio-Rad).

### 2.2. Animal Model

Male Wistar rats (175 to 200 g) were purchased from Charles River Labs (Portage, MI, USA). Rats were randomized into two groups. Animals were weight-matched and randomly assigned to either an ethanol-fed group (ET; n = 7) receiving a Lieber–DeCarli liquid diet containing 6.7% (*v*/*v*) ethanol (Dyets Inc., Bethlehem, PA, USA), or an isocaloric control diet-fed group (CON; n = 8). Rats received an ethanol diet for 6 weeks after a six-day ramp-up period from 0% to 6.7% ethanol diet [11,12]. All animals received humane care in accordance with the guidelines created by the American Association for the Accreditation of Laboratory Animal Care. At study completion, rats were deeply anesthetized with isoflurane. Following confirmation of a surgical plane of anesthesia, the thoracic cavity was opened, blood and VAT samples were collected, and euthanasia was completed by exsanguination and bilateral pneumothorax. Samples were processed appropriately for subsequent analyses. All animal procedures were approved by the Institutional Animal Care and Use Committee (IACUC) of the VA Nebraska-Western Iowa Health Care System (#1616192; approved 28 April 2021).

### 2.3. SVC Isolation

VAT tissues (500 mg/rat) were minced and added to a petri dish with HEPES-buffered DMEM without FBS. Minced tissues were transferred into a 50 mL conical tube and centrifuged at 250× *g* at 4 °C for 10 min to remove erythrocytes. The supernatant containing fat tissue was transferred into a new 50 mL conical tube, and 20 mL HEPES-buffered DMEM containing 1 mg/mL collagenase II and 1% FBS was added for digestion. The tubes were incubated at 37 °C in a rotating incubator for 45 min. After digestion, we filtered the media with minced tissue through a sterile cell strainer (100 μm pore size) into a new 50 mL conical tube. After centrifugation at 500× *g* at 4 °C for 10 min, the supernatant (adipocytes fraction) and pellet (the SVC fraction) were collected. SVC pellets were washed with PBS twice and then used for real-time PCR and Western blot analysis.

### 2.4. Histology

VAT was fixed in 10% formalin. Tissues were embedded in paraffin and sectioned at 9 µm. After deparaffinizing, VAT sections were subjected to hematoxylin and eosin (H&E) staining. As described previously, sections were stained with hematoxylin, followed by rinsing the slides in distilled water three times. After washing the slides with two changes of 70% ethanol for 5 min, the tissue slides were immersed in eosin solution and washed. After dehydration, the slides were mounted using mounting medium. Images were analyzed using AdipoQ, a plugin of ImageJ software (v1.52s, FIJI, NIH, Bethesda, MD, USA), by averaging three randomly selected areas per slide to measure the average adipocyte size and adipocyte number in each VAT slice.

To visualize collagen deposition in VAT sections, VAT sections were subjected to Sirius red staining using the Picro Sirius Red Stain Kit (Ab150681, Abcam, Cambridge, UK), according to the manufacturer’s instructions. Collagen content was quantified using the Image J software (FIJI, NIH, Bethesda, MD, USA) by averaging at least three randomly selected areas per slide. Fields were randomly selected from different regions of each tissue section to obtain representative measurements and minimize sampling bias. The results were presented as the percentage of the total area. Sirius Red-positive collagen was identified using RGB channel separation, followed by threshold-based segmentation, which was then applied unchanged to all images in the analysis.

For immunohistochemistry (IHC), VAT sections were deparaffinized and rehydrated. After antigen retrieval, all sections were incubated with 3% H_2_O_2_ for 10 min and rinsed with distilled water. Next, slides were blocked within 5% bovine serum albumin (BSA) in TBST for 1 h and then incubated in primary antibodies, including antibodies against IL1β, P2RY2, CD45, and COL8A1, at a 1:250 dilution overnight at 4 °C. After washing with fresh TBST, sections were incubated with biotinylated goat anti-rabbit secondary antibody at room temperature for 10 min. After washing with TBST, sections were incubated with streptavidin peroxidase at room temperature, followed by washing with fresh TBST. Then, after color development using diaminobenzidine substrate, VAT sections were counterstained with hematoxylin. A microscope imaging system was used to obtain histology pictures. For each animal, three non-overlapping fields were randomly taken from each adipose tissue slice. Fields were distributed across the section to minimize regional sampling bias. Images were analyzed using ImageJ (Fiji). For quantitative IHC analysis, the tissue area was defined as the total VAT area within each region of interest. Color deconvolution was used to separate the DAB signal from hematoxylin counterstaining, followed by threshold-based segmentation. The threshold established for each marker was applied consistently and without image-specific adjustment to all CON and ET samples. The percentage of positively stained area was calculated by dividing the DAB-positive area by the total VAT area within the region of interest and multiplying by 100.

### 2.5. RNA Sequencing

Total RNA was extracted from the SVCs of CON-fed rats and ET-fed rats (4 per group) using the TRIzol reagent. After assessment of the RNA integrity, RNA sequencing was performed on the Illumina NextSeq 550 system. RNA sequencing was performed using 500 ng of total RNA from each sample using the library kit following the manufacturer’s instructions. The raw data were submitted to the Gene Expression Omnibus database (GSE312640). Raw reads were processed to yield fragments per kilobase of transcript per million mapped reads, and the expression value of each gene was further normalized. Genes with an adjusted *p* < 0.05 were considered significantly altered. Based on significantly altered SVC genes, Ingenuity Pathway Analysis (IPA) was carried out to explore canonical pathways altered due to ethanol exposure. Volcano plots were generated by IPA for one statistical comparison (ET vs. CON). In addition, the Database for Annotation, Visualization and Integrated Discovery (DAVID) Bioinformatics was used to identify signaling pathways related to the role of ethanol in affecting SVC function.

### 2.6. Real-Time PCR

RNA from SVCs was reverse-transcribed utilizing iScript supermix. Real-time PCR was further performed to measure the transcript levels of *P2ry2*, *Col8a1*, *Elovl6*, *Dbp*, *Acsl3*, *Srebf2*, *Npas2*, *Fdft1*, *Rt1-Bb*, and *Rt1-DOa*. The Taqman^TM^ real-time PCR primer probes were used to detect the mRNA levels of genes. Transcript levels were normalized to *Gapdh*. A 2^−ΔΔCT^ method was utilized for the quantification.

### 2.7. Western Blotting

SVC samples were homogenized in RIPA lysis buffer containing phosphatase-protease inhibitor cocktail and centrifuged to collect the supernatants. Proteins were separated by electrophoresis using sodium dodecyl sulfate (SDS)-polyacrylamide gels and immunoblotted to polyvinylidene difluoride membranes. After the transfer, membranes were blocked with 5% BSA for 1 h. After washing three times with TBST, membranes were incubated with primary antibodies overnight at 4 °C. Next, membranes were washed and subsequently incubated with secondary antibody at a 1:5000 dilution for 1 h at room temperature. Secondary antibody signals were revealed by an enhanced chemiluminescence reagent. Protein bands were visualized using the ChemiDoc System (Bio-Rad, Hercules, CA, USA). The total protein level was determined by Coomassie blue staining, which was used for Western blot normalization. Briefly, after protein separation by electrophoresis, SDS-polyacrylamide gels were fixed and stained using Coomassie blue working solution and Coomassie blue staining was visualized by the ChemiDoc System (Bio-Rad).

### 2.8. Statistics

All data are shown as mean ± the standard error of the mean (SEM). Single-factor comparisons were performed by two-tailed Student’s *t*-test. GraphPad Prism 10.1 was used for the statistical analysis. Differences at *p* < 0.05 were considered statistically significant. For RNA-seq, the Benjamini–Hochberg (B-H) method was used to adjust *p* value for multiple-testing-caused false discovery rate (FDR). Adjusted *p* value < 0.05 was considered significant.

## 3. Results

### 3.1. Effects of Ethanol on Increasing VAT Inflammation and Collagen Deposition

**Figure 1:**
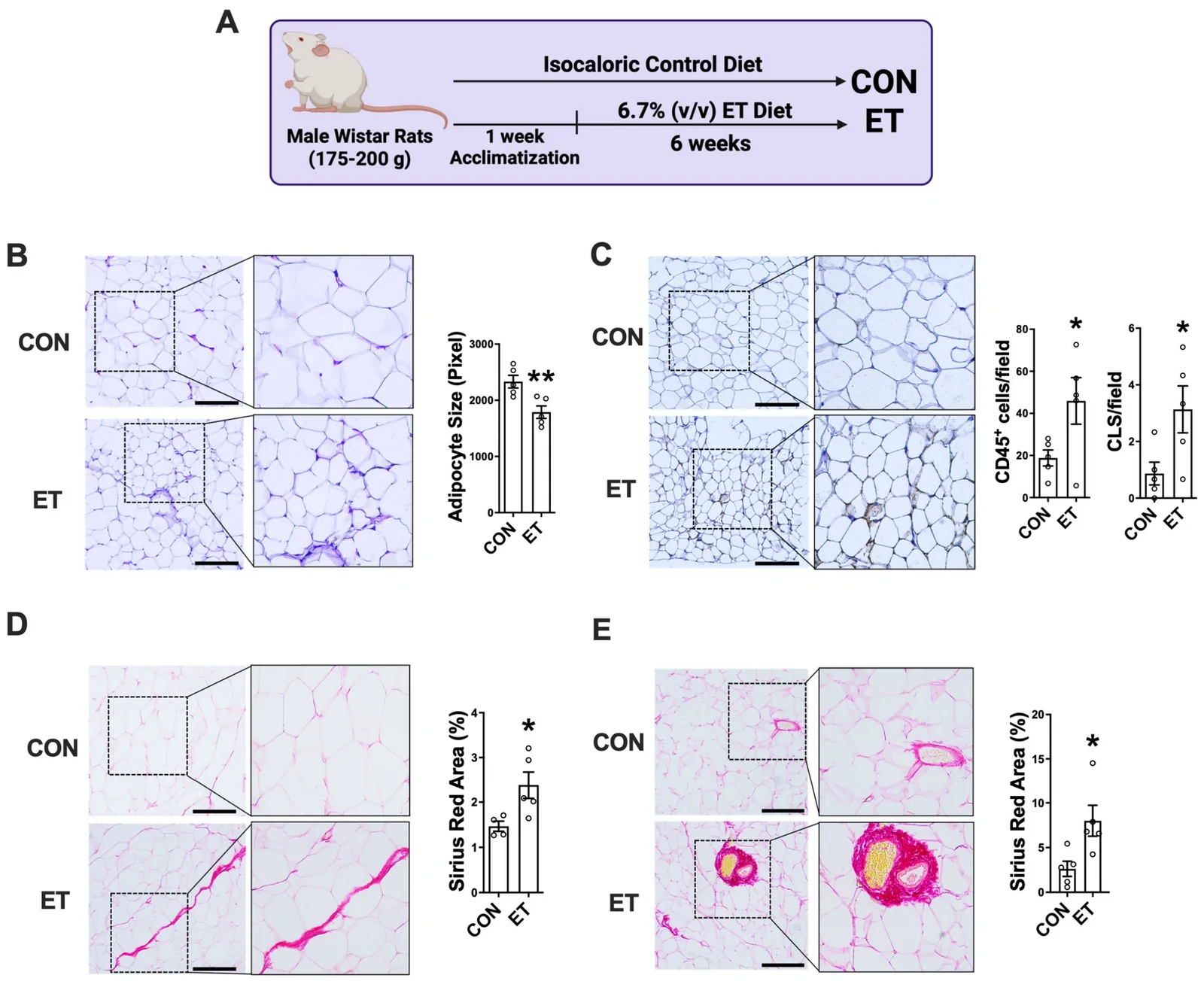
The effects of ethanol on rat VAT. (**A**) Schematic overview for the in vivo experimental design (generated by BioRender). (**B**) Representative pictures of H&E staining (**left**) showing the adipocyte size of VAT in rats fed ET and CON for six weeks, scale bar = 100 μm. Quantification of adipocyte size (**right**), n = 5 per group. (**C**) Representative pictures of IHC for CD45-positive cells (**left**) in VAT of ET- and CON-fed rats, scale bar = 100 μm. Quantification of the number of CD45-positive cells and CLS (**right**), n = 5 per group. (**D**) Representative pictures (**left**) and quantification (**right**) of Picro Sirius Red staining exhibiting interstitial collagen deposition in VAT sections from each group, scale bar = 100 μm, n = 4–5 per group. (**E**) The representative pictures (**left**) and quantification (**right**) of Picro Sirius Red staining exhibiting collagen deposition in perivascular regions of VAT sections from each group, scale bar = 100 μm, n = 5 per group. Values are presented as mean ± SEM, * *p* < 0.05, ** *p* < 0.01 vs. CON. Statistical analyses were performed using two-tailed Student’s *t*-test.

To investigate the effects of ET in causing VAT dysfunction, male Wistar rats were fed an ethanol diet or a CON diet for six weeks (Figure 1A). Histological analysis showed a reduction in adipocyte size in VAT from ET-fed rats compared with CON rats (*p* < 0.05, Figure 1B), indicating ET-induced impairment in lipid storage function of AT. Next, IHC was performed to show the number of immune cells (CD45-positive cells) and the crown-like structures (CLS), the histological marker for chronic AT inflammation, in VAT. We noted that ET-fed rats showed an increased number of immune cells (*p* < 0.05, Figure 1C) and CLS (*p* < 0.05, Figure 1C) in VAT, indicating ET-induced immune cell infiltration and inflammation.

To determine whether chronic ethanol exposure was also associated with AT remodeling, rat VAT sections were subjected to Picro Sirius Red Staining. ET-fed rats showed an increase in collagen deposition in VAT compared with CON-fed rats (*p* < 0.01, Figure 1D). Interestingly, ET-fed rats exhibited a marked increase in collagen accumulation within the perivascular area of VAT (Figure 1E), indicating the adverse effects of chronic alcohol exposure in promoting collagen deposition in rat VAT.

### 3.2. Transcriptomic Profiling of VAT-Derived SVCs Identifies Pathways Associated with Ethanol-Induced VAT Dysfunction

**Figure 2:**
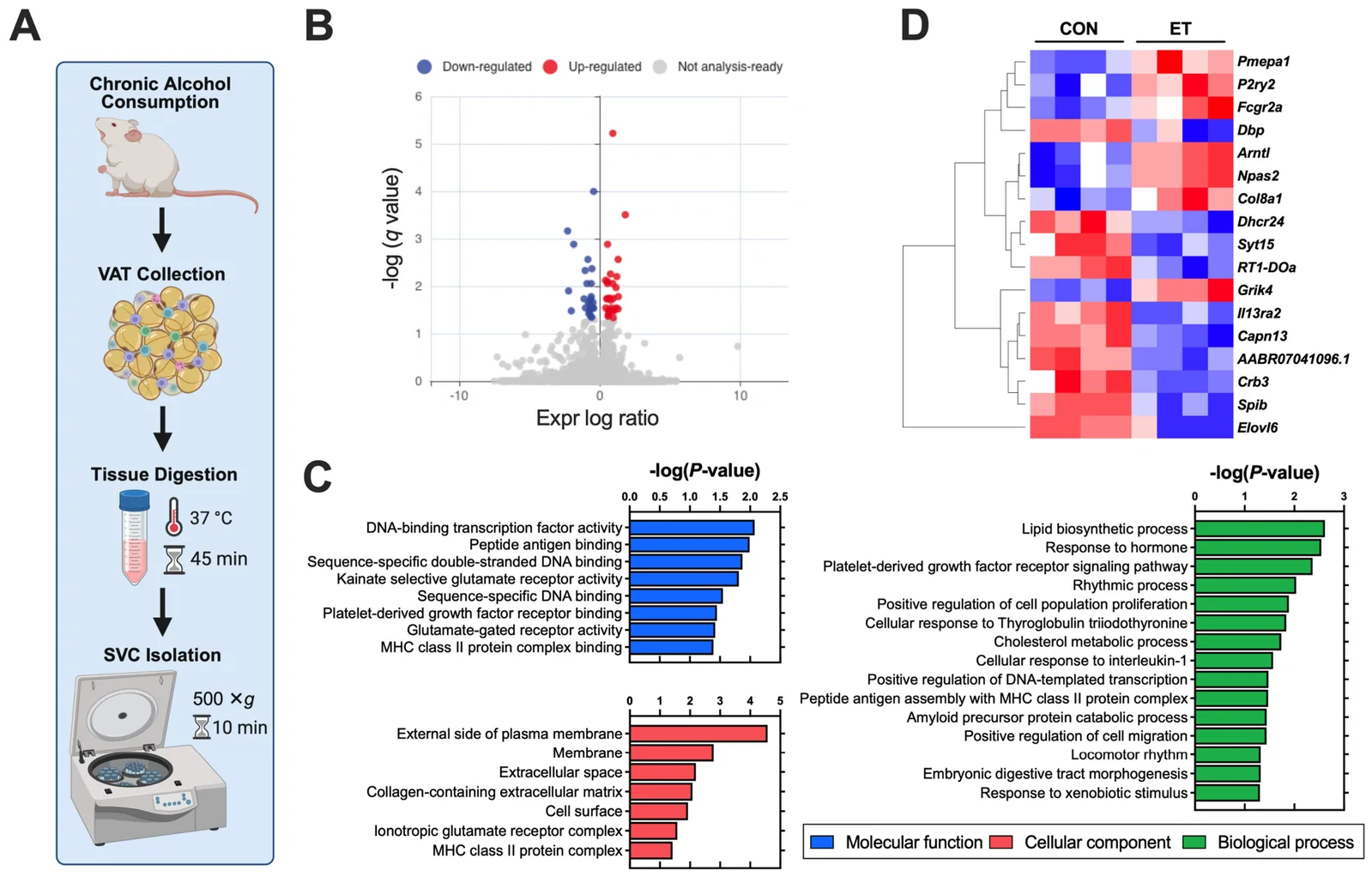
Transcriptional profiling of SVCs in VAT of ET-fed rats. (**A**) Schematic overview for SVC isolation (generated by BioRender). (**B**) Volcano plot showing differentially expressed genes in response to chronic alcohol exposure, n = 4 rats per group. Differentially expressed genes were identified after adjustment for multiple testing using the Benjamini-Hochberg procedure, and genes with an adjusted *p* value < 0.05 were considered statistically significant. (**C**) Altered signaling pathways in SVCs in response to chronic ethanol exposure, as determined via the DAVID database. (**D**) A heatmap of the representative differentially expressed genes identified by RNA-seq analysis, based on the cutoff (adjusted *p* < 0.05 and log2 (fold change) ≤ −1 or ≥1); red represents upregulated expression, while blue represents downregulated expression.

Since SVCs mediate AT inflammation and fibrosis, we next sought to determine the molecular alterations in SVCs associated with ethanol-induced VAT dysfunction. Primary SVCs were isolated from VAT of CON and ET-fed rats (Figure 2A). Next, we performed RNA-sequencing analysis and identified 57 genes that were significantly altered in the SVCs of rats receiving a chronic ethanol diet compared to a control diet (Figure 2B). The gene ontology (GO) analysis suggests that the significantly altered genes were enriched in pathways related to major histocompatibility complex (MHC) class II protein binding, lipid biosynthetic process, rhythmic process, cellular response to IL-1, and collagen-containing extracellular matrix (Figure 2C).

To further evaluate the broader transcriptomic profile identified by RNA-seq, we examined representative genes associated with MHC class II protein binding and lipid biosynthesis. Among the MHC class II-related genes, *Rt1-Bb* expression was significantly decreased in the total VAT-derived SVCs from ET-fed rats, whereas *Rt1-DOa* expression was not significantly altered (Supplementary Figure S1). We also evaluated the lipid biosynthesis-related genes, including *Acsl3*, *Srebf2*, *Npas2*, and *Fdft1*. Although these genes were significantly altered in the RNA-seq dataset, their expression differences were not independently confirmed by qPCR in the larger cohort (Supplementary Figure S1). Thus, in contrast to the RNA-seq results, these findings did not provide evidence for chronic alcohol-induced alterations in MHC class II and lipid biosynthesis pathways.

Interestingly, among those altered genes, *P2ry2* and *Col8a1* were closely linked to inflammatory and extracellular matrix-related pathways, respectively (Figure 2D). These transcriptomic findings suggest that chronic ethanol exposure is associated with changes in SVC gene expression patterns relevant to inflammatory signaling and remodeling in VAT.

### 3.3. Chronic Alcohol Exposure Is Associated with Increased P2RY2-Related Inflammatory Signaling in VAT and SVCs

**Figure 3:**
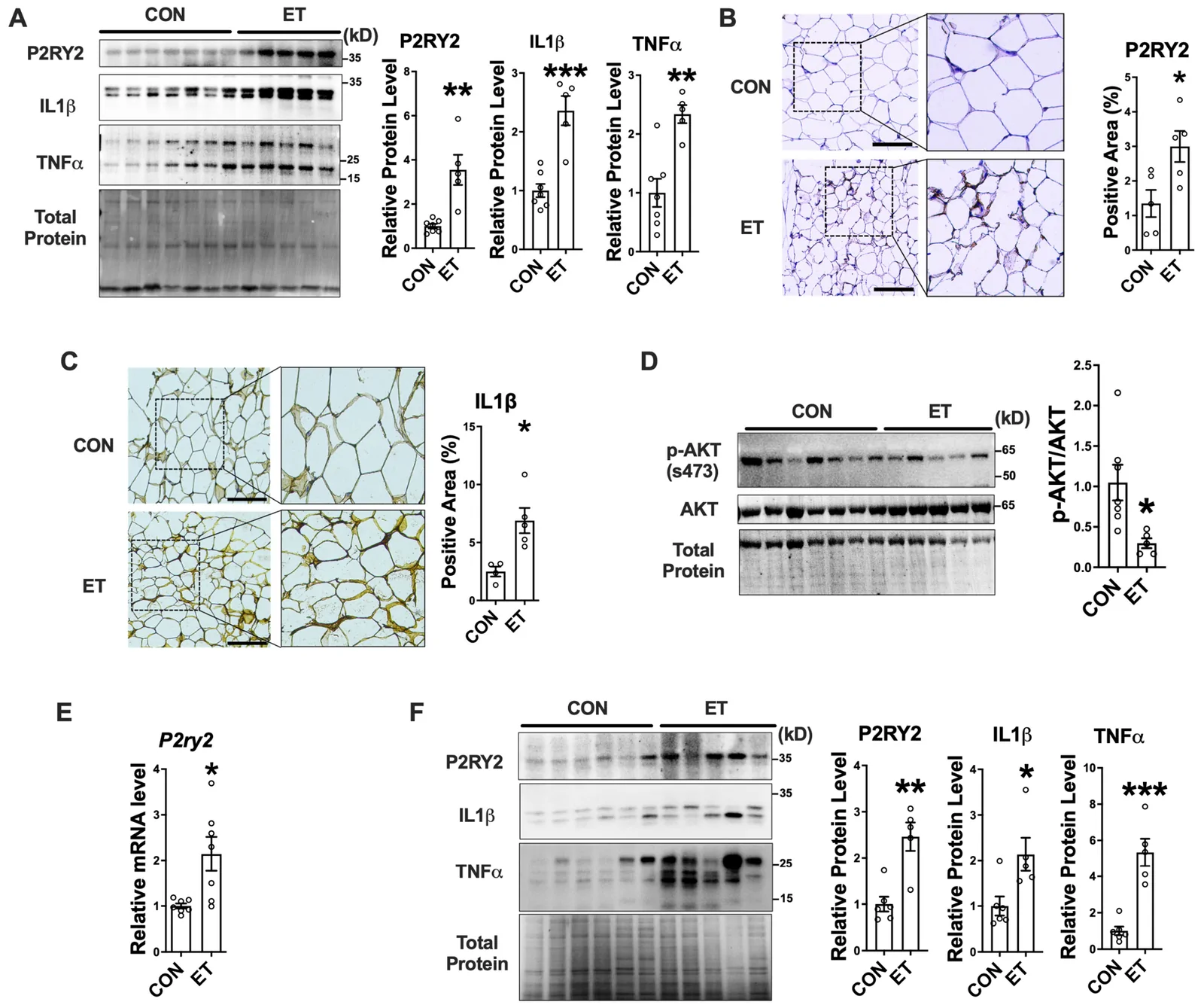
The effect of ethanol on P2RY2 expression and markers of inflammation and/or insulin resistance in VAT and SVCs. (**A**) Representative Western blot images (**left**) and densitometric analysis of the band intensities (**right**) for P2RY2, IL1β, and TNFα in VAT of rats fed ET and CON diets for six weeks, n = 5–7 per group. (**B**) Representative pictures of IHC (**left**) and quantification (**right**) for P2RY2 expression in the VAT of rats fed ET and CON diets for six weeks, scale bar = 100 μm, n = 5 per group. (**C**) Representative pictures of IHC (**left**) and quantification (**right**) for IL1β expression in VATs of ET and CON rats, scale bar = 100 μm, n = 4−5 per group. (**D**) Representative Western blot images (**left**) and densitometric analysis of the band intensities (**right**) for p-AKT and AKT in VAT of ET- and CON-fed rats, n = 5–7 per group. (**E**) Real-time PCR analysis showing transcript levels for *P2ry2* of SVCs of ET and CON rats, n = 7 per group. (**F**) Western blot images (**left**) and densitometric analysis of the band intensities (**right**) for P2RY2, IL1β, and TNFα in SVCs of ET- and CON-fed rats. n = 5–6 per group. Values are normalized to total protein. Values are reported as mean ± SEM. * *p* < 0.05, ** *p* < 0.01, *** *p* < 0.001 vs. CON. Statistical analyses were performed using two-tailed Student’s *t*-test.

P2ry2 was among the top 25 differentially expressed genes when ranked by adjusted *p* value and was linked by GO analysis to the “cellular response to interleukin-1” pathway in SVCs (Figure 2C,D). Real-time PCR, Western blot, and IHC were next performed to validate RNA-seq results. In the whole VAT tissue, Western blot analysis showed that chronic alcohol exposure led to increased protein level of P2RY2 (*p* < 0.01, Figure 3A). Given that P2RY2 regulates IL1β and TNFα expression in AT [13,14], we further examined whether ethanol exposure affects the production of pro-inflammatory mediators in VAT. We found that the protein levels of IL1β (*p* < 0.001) and TNFα (*p* < 0.01) were increased in VAT (Figure 3A). In line with these results, immunohistochemical analysis revealed an increase in P2RY2 staining (*p* < 0.05, Figure 3B) in the VAT of ET-fed rats compared with control diet-fed rats. Moreover, ET diet-fed rats displayed higher IL1β staining in VAT compared with controls (*p* < 0.05, Figure 3C), further supporting the role of alcohol in leading to increased inflammatory response in the VAT tissue. There is a tight link between P2RY2 and impaired insulin signaling in AT [15]. ET-fed rats showed a decrease in the phosphorylation of protein kinase B (AKT), a key kinase mediating the insulin signaling cascade, in VAT (*p* < 0.05, Figure 3D).

To determine whether these alcohol-induced adverse alterations were also shown in the stromal vascular cells, we measured P2RY2 and pro-inflammatory mediators in isolated SVCs. ET-fed rats exhibited an upregulation of the mRNA level of *P2ry2* in SVCs compared with CON-fed rats (*p* < 0.05, Figure 3E). Moreover, the protein levels of P2RY2 (*p* < 0.01), IL1β (*p* < 0.05), and TNFα (*p* < 0.001) were increased in SVCs of VAT upon ethanol feeding (Figure 3F). Together, these findings indicate that chronic ethanol exposure is associated with increased P2RY2 expression and inflammatory mediator production in both whole VAT and VAT-derived SVCs.

### 3.4. Chronic Alcohol Exposure Is Associated with Increased Perivascular Collagen Type VIII Deposition and Activation of TGFβ Signaling Pathway in VAT

**Figure 4:**
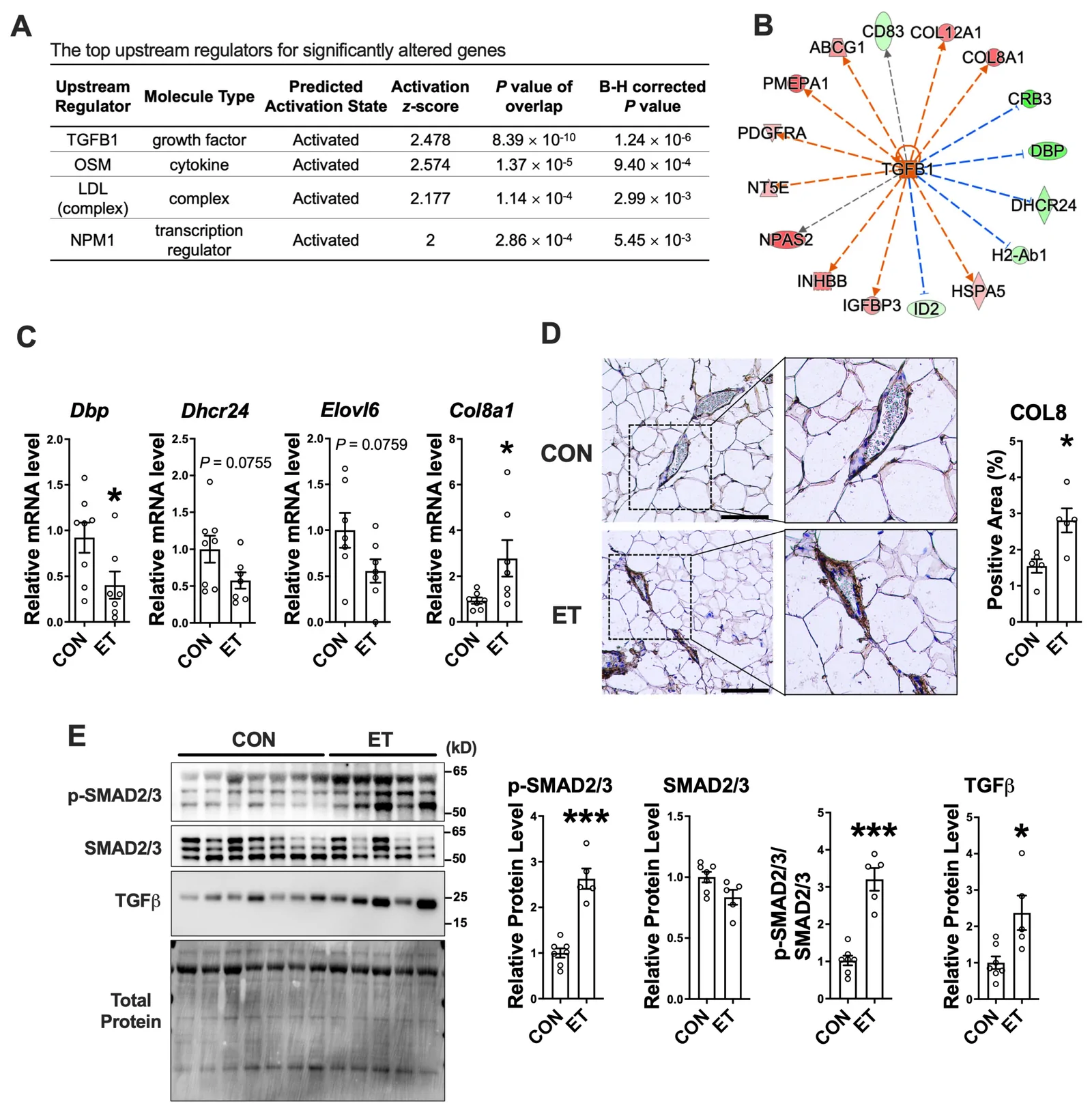
The effect of ethanol on TGFβ signaling in SVCs and VAT and markers of collagen deposition in VAT. (**A**) Top biological upstream regulators identified by IPA. (**B**) The upstream regulatory network of TGFβ. (**C**) Real-time PCR analysis showing transcript levels of TGFβ target genes, *Dbp*, *Dhcr24*, *Elovl6*, and *Col8a1*, in SVCs of ET- and CON-fed rats, n = 7–8 per group. (**D**) Representative pictures of IHC (**left**) and quantification (**right**) for COL8A1 expression in the perivascular region of VAT in ET- and CON-fed rats, scale bar = 100 μm, n = 5 per group. (**E**) Western blot images (**left**) and densitometric analysis of the band intensities (**right**) for phosphorylated SMAD2/3, total SMAD2/3, and TGFβ in the VAT of ET- and CON-fed rats. Values are normalized to total protein, n = 5–7 per group. Values are expressed as mean ± SEM, * *p* < 0.05, *** *p* < 0.001 vs. CON. Statistical analyses were performed using two-tailed Student’s *t*-test.

To identify pathways potentially involved in ethanol-associated VAT remodeling, we performed the IPA analysis of the differentially expressed genes between CON- and ET-fed groups, which revealed the top upstream regulators in SVCs (Figure 4A), including TGFβ, oncostatin M (OSM), low-density lipoprotein (LDL), and nucleophosmin 1 (NPM1). Upstream regulator analysis further identified TGFβ as a predicted upstream regulator (Figure 4B, z-score = 2.478, adjusted *p* < 0.0001) associated with ethanol exposure. Genes within this predicted network included *Col8a1*, *Dbp*, and *Dhcr24*. To evaluate selected genes from this predicted network, real-time PCR was carried out, which indicated downregulated mRNA levels of *Dbp* (*p* < 0.05), *Dhcr24* (*p* = 0.0755), and *Elovl6* (*p* = 0.0759), and an upregulated mRNA level of *Col8a1* (*p* < 0.05) in SVCs from rats upon ethanol exposure (Figure 4C).

Collagen type VIII, a short-chain and non-fibrillar collagen, is predominantly localized within the vasculature of AT. To further characterize collagen type VIII distribution in rat VAT, we performed IHC, which demonstrated that COL8A1 expression was primarily present in the perivascular region (Figure 4D). Notably, rats fed a chronic ethanol diet exhibited an increase in COL8A1 deposition in the perivascular area compared with controls (Figure 4D). To further assess whether the TGFβ pathway is activated, protein levels of phosphorylated SMAD2/3 and TGFβ were measured in VAT. ET-fed rats showed an increase in the protein levels of phosphorylated SMAD2/3 (*p* < 0.001, Figure 4E) and TGFβ (*p* < 0.05, Figure 4E) in VAT, indicating an activation of the TGFβ signaling pathway in VAT caused by chronic alcohol exposure.

Taken together, these findings suggest that chronic ethanol exposure is associated with increased TGFβ/SMAD-related signaling markers, collagen accumulation, and COL8A1 deposition in the perivascular region of VAT.

## 4. Discussion

In the present study, we characterized pathological and molecular alterations in VAT from rats fed a chronic alcohol-containing diet. ET-fed rats exhibited an increase in immune cell infiltration and CLS formation in the VAT. Primary SVCs were isolated from VAT of CON- and ET-fed rats for RNA-seq, which identified pathways related to inflammatory signaling and extracellular matrix remodeling that were significantly altered in SVCs from ET-fed rats. Notably, chronic alcohol exposure led to increased production of pro-inflammatory mediators, including TNFα and IL1β, which is associated with an increased protein level of P2RY2 in the whole VAT and isolated SVCs. In addition, chronic alcohol exposure triggered excessive collagen deposition in both interstitial and perivascular regions of VAT, along with activation of the TGFβ-SMAD axis. Specifically, COL8A1 expression, regulated by TGFβ signaling pathway, was upregulated in the perivascular niche of VAT. These findings suggest that chronic ethanol exposure is associated with inflammatory and remodeling-related changes in VAT and VAT-derived SVCs. In particular, P2RY2 and TGFβ emerged as candidate players mediating VAT inflammation and remodeling, respectively, upon ethanol exposure.

Chronic alcohol consumption is known to impair AT storage function, and the present findings add further evidence that VAT is an important target of ethanol-induced tissue injury. Notably, histological analyses from the present study showed that chronic alcohol exposure decreased adipocyte size, increased immune cell infiltration and the generation of pro-inflammatory cytokines, and promoted the formation of CLS, a special pathological structure that is composed of dead or dying adipocytes and surrounding macrophages. CLS is the hallmark of pro-inflammatory lesions in AT. Several studies using models of metabolic dysfunction-associated steatotic liver disease (MASLD) have shown that mice with obesity displayed an increased number of CLS and a higher production of pro-inflammatory mediators, including tumor necrosis factor-α (TNF-α), IL1β, inducible nitric oxide synthase (iNOS), and hypoxia inducible factor 1 subunit α in AT [16,17]. These findings suggest that chronic ethanol exposure led to abnormal adipocyte morphology and the inflammatory microenvironment in VAT.

A major objective of this study was to determine the role of SVCs in ethanol-associated VAT dysfunction. SVCs are composed of multiple non-adipocyte cell populations, including immune cells, endothelial cells, fibroblasts, and adipose progenitor cells, all of which can play an important role in AT inflammation and remodeling. In the present study, RNA-seq analysis of isolated VAT-derived SVCs identified differentially expressed genes enriched in signaling pathways related to the cellular response to IL-1 and collagen-containing extracellular matrix. These findings broaden the understanding of VAT dysfunction in ALD by showing a chronic ethanol exposure-induced transcriptional profile in SVCs, supporting a critical role for non-adipocyte cell populations in VAT inflammation and remodeling during chronic alcohol exposure. Among those significantly altered genes of SVCs, ET-fed rats showed increased *P2ry2* mRNA levels along with increased pro-inflammatory cytokine production in SVCs. P2RY2 is a G protein-coupled purinergic receptor that is activated by ATP and UTP and widely expressed in AT. In MASLD, which is characterized by increased CLS formation in ATs, extracellular ATP was released from dead or dying adipocytes, promoting a purinergic receptor-mediated inflammatory response [18]. In particular, P2RY2 acts as a regulator of inflammatory response in diverse diseases [19,20]. For example, mice exposed to high fat diet-feeding for 14 weeks displayed increased P2RY2 expression in white AT, and globally knocking out *P2ry2* exerted protective effects in attenuating high-fat diet-induced AT inflammation [21]. In ALD studies, P2RY2 aggravated alcohol-induced inflammatory response in the liver by targeting the mitogen-activated protein kinase (ERK) signaling pathway [19]. These findings suggest a strong link between P2RY2 and inflammatory response in alcohol-induced VAT dysfunction.

In addition to ethanol-induced inflammation in AT, the present study has shown a reduction in the phosphorylation of AKT, a key kinase mediating insulin signaling transduction, in VAT from ET-fed rats. In metabolic syndrome, pro-inflammatory cytokines play an important role in provoking insulin resistance [22]. More interestingly, several lines of studies have shown the tight link between P2RY2 and insulin signaling transduction. An in vivo study has reported that global knockout of P2RY2 attenuates high-fat diet-induced adipogenesis, inflammation, and aberrant lipid metabolism in white AT, thereby mitigating systemic insulin resistance [21]. A mechanistic study from Qian et al. using human primary visceral preadipocytes has shown that activation of P2RY2 by UTP treatment led to increased phosphorylation of ERK and C-Jun N-terminal kinase 1 (JNK) and decreased phosphorylation of AKT, leading to compromised insulin sensitivity [15]. These findings highlight adipocyte P2RY2 as a potential therapeutic target in obesity and type 2 diabetes. In terms of ALD, chronic alcohol exposure induces white AT dysfunction by elevating the secretion of pro-inflammatory cytokines and adipocyte apoptosis and compromising insulin-dependent glucose uptake, contributing to systemic insulin resistance [23,24]. Our findings are consistent with a potential relationship between ethanol-associated inflammatory signaling in SVCs and altered markers of insulin signaling transduction in VAT. However, additional studies will be warranted to determine whether P2RY2 signaling in VAT and/or SVCs directly contributes to impaired VAT insulin signaling during chronic ethanol exposure.

Another important finding of this study is that chronic alcohol consumption induced excessive collagen deposition in the perivascular region of VAT and identified *Col8a1* as an ethanol-responsive gene in SVCs. It is well-known that chronic or heavy alcohol exposure elicits tissue remodeling [25,26]. Both preclinical and clinical studies have shown that ALD is characterized by excessive collagen deposition in the liver by promoting the synthesis of extracellular matrix proteins from the activated hepatic stellate cells, a type of myofibroblast-like cell, in the liver [26]. Specifically, the TGFβ signaling pathway exerts profound effects on promoting ALD progression by increasing inflammation and fibrosis and inhibiting hepatocyte regeneration [27]. Interestingly, RNA-seq from the present study identified upregulation of TGFβ signaling in SVCs from ET-fed rats. In addition, we provide novel findings that the TGFβ signaling pathway was activated in VAT, as evident from an increase in the protein level of TGFβ and phosphorylated SMAD2/3, which were associated with alterations in the expression of its target genes, including *Dbp* and *Col8a1.* More interestingly, we found an increased collagen deposition in the perivascular region of VAT in chronic ET-fed rats. Collagen type VIII, a short-chain and non-fibrillar collagen, is mainly produced by endothelial cells or pre-adipocytes and is present in the vasculature. Clinical studies have demonstrated that aberrant upregulation of collagen VIII is implicated in the pathogenesis of a broad spectrum of diseases, including diabetic nephropathy, chronic obstructive pulmonary disease, and cancers [28,29]. Collagen type VIII has emerged as a regulator of maladaptive remodeling and tumor progression [30]. Therefore, these findings indicate that chronic alcohol exposure is associated with VAT remodeling, accompanied by increased TGFβ/SMAD-related signaling markers and perivascular COL8A1 deposition.

There are some limitations to this study. For example, this pilot study identifies associations between chronic ethanol exposure and molecular changes in P2RY2-associated inflammatory markers and TGFβ-related remodeling markers, but it does not establish direct causality. Pharmacological interventions, gene-silencing approaches, or genetic animal models will be required to determine whether P2RY2 and TGFβ signaling functionally contribute to inflammatory and pro-fibrogenic responses in VAT-derived SVCs following chronic ethanol exposure. Additionally, the relatively small sample size, particularly for the RNA-seq analysis, limits the statistical power and generalizability of this exploratory pilot study. Future studies using larger independent cohorts will be needed to validate the broader transcriptomic profile. Moreover, because the SVC contains multiple cell types, the bulk RNA-seq and Western blot analyses cannot identify the specific cell population responsible for the observed molecular changes caused by alcohol exposure. These changes may reflect altered expression within individual cell types, shifts in SVC composition, or both. Accordingly, our findings are interpreted as ethanol-associated changes in the collective stromal vascular compartment. Future studies using flow cytometry, computational deconvolution, or single-cell RNA-seq will be required to characterize ethanol-associated alterations in SVC composition and identify the specific cell type responsible for observed molecular changes.

## 5. Conclusions

The present exploratory pilot study shows that chronic alcohol exposure is associated with pathological alterations in rat VAT, including increased immune cell infiltration, CLS formation, and collagen deposition in interstitial and perivascular regions. Transcriptomics and protein analyses of the total VAT-derived SVC fraction identified ethanol-associated alterations in P2RY2-related inflammatory markers and TGFβ-related remodeling markers. Taken together, these findings broaden our understanding of alcohol-associated VAT dysfunction beyond adipocytes alone and identify candidate inflammatory and remodeling pathways for future cell-type-resolved and mechanistic studies.

## Data Availability

The raw data supporting the conclusions of this article will be made available by the authors on request.

## Supplementary Materials

The following supporting information can be downloaded at: https://www.mdpi.com/article/10.3390/biom16101440/s1, Supplementary Figure S1: Real-time PCR analysis showing transcript levels for *Rt1-Bb*, *RT1-DOa*, *Acsl3*, *Srebf2*, *Naps2*, and *Fdft1* of SVCs of ET and CON rats.

## Abbreviations

AKT	Protein kinase B
ALD	Alcohol-associated liver disease
AT	Adipose tissue
BSA	Bovine serum albumin
CD45	leukocyte common antigen
CLS	Crown-like structures
CON	Isocaloric control diet
COL8	Collagen type VIII
COL8A1	Collagen type VIII alpha 1 chain
DAVID	Database for annotation, visualization and integrated discovery
DBP	D-box binding PAR BZIP transcription factor
DHCR24	24-dehydrocholesterol reductase
DMEM	Dulbecco’s modified eagle’s medium
ECM	Extracellular matrix
ELOVL6	ELOVL fatty acid elongase 6
ERK	Mitogen-activated protein kinase
ET	Ethanol-containing Lieber–DeCarli diet
FBS	Fetal bovine serum
GAPDH	Glyceraldehyde-3-phosphate dehydrogenase
GO	Gene ontology
H&E	Hematoxylin and eosin
IHC	Immunohistochemistry
IL1	Interleukin 1
iNOS	Inducible nitric oxide synthase
IPA	Ingenuity pathway analysis
JNK	C-Jun N-terminal kinase 1
LDL	Low-density lipoprotein
MASLD	Metabolic dysfunction-associated fatty liver diseases
MHC	Major histocompatibility complex
NPM1	Nucleophosmin 1
OSM	Oncostatin M
P2RY2	Purinergic receptor P2Y2
SEM	Standard error of the mean
SMAD	SMAD family member
SVC	Stromal vascular cells
TBST	Tris-buffered saline with Tween-20
TGFβ	Transforming growth factor β
TNFα	Tumor necrosis factor α
VAT	Visceral adipose tissue
