# Supplementary material for: Stromal Vascular Cell-Mediated Inflammatory and Remodeling Pathways in Alcohol-Induced Visceral Adipose Tissue Dysfunction: A Pilot Study
Source: Biomolecules. 2026 Oct 3;16(10):1440. doi: 10.3390/biom16101440 (PMC13702598; doi:10.3390/biom16101440)
Supplement: Supplementary file 1 [file biomolecules-16-01440-s001.zip › biomolecules-4471932-supplementary.pdf]

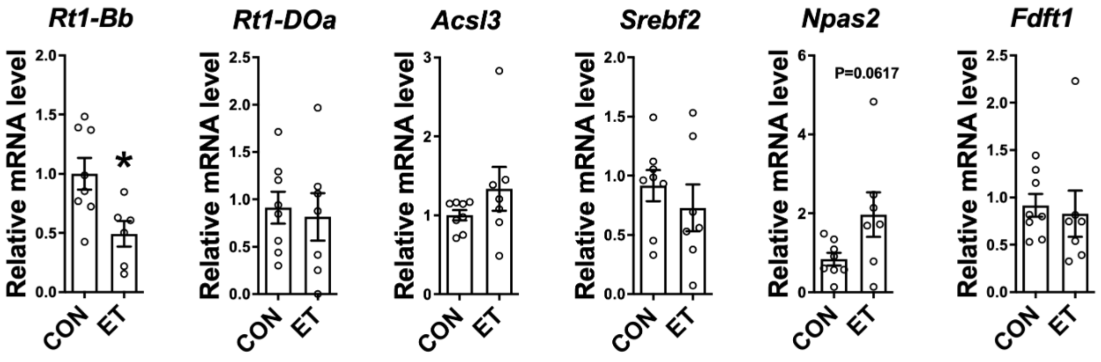

Supplementary Figure S1. Real-time PCR analysis showing transcript levels for *Rt1-Bb*, *RT1-DOa*, *Acsl3*, *Srebf2*, *Naps2*, and *Fdft1* of SVCs of ET and CON rats, n = 7 per group.
